# The use of patient-reported outcome measures in hip and knee arthroplasty in Alberta

**DOI:** 10.1186/s41687-021-00362-6

**Published:** 2021-10-12

**Authors:** Deborah A. Marshall, Xuejing Jin, Lindsay B. Pittman, Christopher J. Smith

**Affiliations:** 1 Department of Community Health Sciences, Cumming School of Medicine, University of Calgary Health Research Innovation Centre – 3C56, 3280 Hospital Drive NW, Calgary, AB T2N 4Z6 Canada; 2 Centre for Evidence-Based Medicine, School of Traditional Chinese Medicine, Beijing University of Chinese Medicine, No. 11, Bei San Huan Dong Lu, Chaoyang District, Beijing, 100029 China; 3 Alberta PROMs & EQ-5D Research & Support Unit, School of Public Health, University of Alberta, 11405 87 Avenue, Edmonton, AB T6G 1C9 Canada; 4 Alberta Bone and Joint Health Institute, Suite 316, 400 Crowfoot Crescent NW, Calgary, AB T3G 5H6 Canada

**Keywords:** Patient-reported outcome measures (PROMs), Patient-reported experience measures (PREMs), Hip and knee arthroplasty, Appropriateness, Decision aid, Key performance indicators

## Abstract

PROMs are part of routine measurement for hip and knee replacement in Alberta, Canada. We provide an overview of how PROMs are implemented in routine care, and how we use PROMs data for decision-making at different levels within the health system. The Alberta Bone and Joint Health Institute (ABJHI) ran a randomized controlled trial to determine the effectiveness and cost-effectiveness of an evidence-based care pathway for hip and knee arthroplasty in 2004. The study included several PROMs questionnaires: Western Ontario and McMaster Universities Osteoarthritis Index, Health Utility Index, Short Form 36 and the EQ-5D-3L. Subsequently, the focus shifted to spread and scale of the care pathway provincially. WOMAC and EQ-5D-3L and a patient experience survey were selected for provincial adoption – captured before surgery, three-months post-surgery, and 12-months post-surgery. These PROMs data were integrated into research and routine clinical practice at the micro, meso and macro levels. At the micro level, PROMs data are used at the individual patient and provider level for patients to provide input on their care and as a tool to communicate with their healthcare providers. We examined the relationship of appropriateness and patient reported outcomes in a prospective cohort study. We evaluated whether routinely collected PROMs could be integrated into a patient decision aid to better inform shared decision making. At the meso level, continuous quality improvement reports are provided routinely to individual health care providers, hospitals and clinics on their performance against the measurement framework and standard key performance indicators. At the macro level, PROMs data are used to evaluate system performance by comparing outcomes across different jurisdictions or over time and support health policy decision making. Combined with administrative databases, we have used simulation models to reflect transition through the continuum of care from disease onset through end-stage care regarding the burden of disease, healthcare resource requirements and associated healthcare costs. The addition of PROMs data in clinical repositories and analyses enables the system to identify and address issues of continuous quality improvement against a measurement framework of performance indicators and to explicitly recognize the trade-offs that are inherent in any resource-constrained system.

## Background

In the last two decades, the number of joint replacements grew rapidly around the world [1]. Patient-reported outcomes following joint replacement have been considered an important indicator of evaluating the outcome of these procedures, with the increasing orientation towards patient-centred care. Patient-reported outcome measures (PROMs) have been routinely applied in hip and knee replacement programs in the United Kingdom and Sweden [2, 3]. The Organization for Economic Co-operation and Development (OECD) is also piloting its Patient-Reported Indicator Surveys initiative in the field of hip and knee replacement to promote a consistent approach across OECD countries to collect PROMs data [4].

Likewise, implementation of PROMs has also become part of the routine care for hip and knee replacement in Alberta, Canada. This paper first introduces briefly how PROMs are implemented in routine care, how we use PROMs data to make decisions at different levels within the health system, and the associated challenges with implementing PROMs with examples of what have we learned from implementation efforts.

## PROMs implementation (Table 1)

**Table 1:** Summary of hip and knee arthroplasty PROMs implementation phases in Alberta

Phase (fiscal year end)	TJA surgeries (#)	PROMS captured (#, %)†	Participating hospitals (#, %)††	Participating physicians (#, %)†††
Pilot study (2004–2006)	18,862	3,447 (18%)	7 (54%)	20 (31%)
Spread and scale (2006–2011)	27,154	6,688 (25%)	9 (64%)	31 (41%)
Continuous quality improvement (CQI) reporting (2011–2020)	87,890	56,086 (64%)	14 (100%)	84 (100%)

^†^Includes all surgical patients who completed both a pre-surgery and 3-month post-surgery PROMs survey

^††^Includes all hospitals who perform elective hip and knee arthroplasty in Alberta

^††^† Includes all physicians who performed at least 10 arthroplasty surgeries per year

The application of PROMs in hip and knee arthroplasty in Alberta began in 2004, when the Alberta Bone and Joint Health Institute (ABJHI) ran a randomized controlled trial sponsored by Alberta Health and Wellness, the Alberta Orthopedic Society and three regional health authorities (Calgary Health Region, Capital Health, and David Thompson Health Region) [5]. The two-year study determined the effectiveness and cost-effectiveness of a novel, evidence-based care pathway for hip and knee replacement [6]. Central to that effort was the measurement of several PROMs to capture health status and health utility. The study included several PROMs questionnaires including the Western Ontario and McMaster Universities Osteoarthritis Index (WOMAC) [7], Health Utility Index Mark 3 (HUI3) [8], Short Form 36 (SF-36) [9] and the EQ-5D-3L [10].

Following the successful conclusion of the study, the focus shifted to spread the pathway across the rest of the province and to scale up data capture to all patients seeking care. With the transition to routine care, the implementation team selected the WOMAC and EQ-5D-3L along with a non-validated patient experience survey for provincial adoption. Among the set of instruments captured during the study, these PROMs were deemed the most clinically and operationally meaningful, the least redundant, and the easiest to implement at a large scale. The WOMAC and EQ-5D were captured on paper before surgery, three months post-surgery, and 12-months post-surgery, and scanned using optical character recognition (OCR) technology into a database for statistical analysis and reporting.

## Summary of the up-to-date PROMs data repository

Following completion of the spread and scale phase in 2011, ABJHI introduced automated reporting and feedback systems, electronic capture of PROMs, and transitioned from the EQ-5D-3L to the EQ-5D-5L [11]. The paper-based capture of PROMs proved to be time-consuming, expensive and prone to errors. As the volume of patients and participating clinics increased, the corresponding volume of paper soon exceeded 30,000 pages per year, costing about $20,000 in labour. OCR errors on the patient labels confounded patient identification, which required significant effort to correct. Furthermore, the process of collecting, collating, transporting, scanning, validating, and integrating PROMs data from paper into the database often resulted in delays of up to six months between data capture and the ability to analyze and report results.

Electronic capture was implemented using two data capture platforms that were integrated into the clinic workflows. Some clinics had licensed and implemented the proprietary Ortech software [12] to record orthopedic implant use and to submit data to the Canadian Joint Replacement Registry. These clinics opted to implement PROMs capture using Ortech. For the other clinics, ABJHI provided an open-source solution using REDCap software [13].

The EQ-5D-5L was introduced because it was considered superior to the 3L version due to 1) the increase in possible health states from 243 to 3,125 which offered better detection in changes in health-related quality of life, 2) the publication of Canadian value sets in the 5L provided more regionally relevant scoring than was available in the 3L, and 3) the 5L score was believed to be less sensitive to a ceiling effect when measuring the impact of arthritis on quality of life. We used PROMs data collected in this repository to compare the cross-sectional and longitudinal validity of the EQ-5D-3L and EQ-5D-5L. The results demonstrated that the EQ-5D-5L has better construct validity and responsiveness (tested against the WOMAC) than the 3L version. These findings also supported our decision to replace the EQ-5D-3L with the EQ-5D-5L as a PROM for routine data collection [14, 15].

## Application of PROMS in routine clinical practice and health care services

In this section, we highlight some novel applications of these PROMs data in research programmes and integrated into routine clinical practice settings to deliver healthcare services and to assess patient outcomes and performance of the system at the micro, meso and macro levels in the healthcare system in Alberta [16].

At the micro level, PROMs data can be used at the individual patient and provider level for patients to provide input on their care and as a tool to communicate with their healthcare providers. An example is the development of a mobile health app for patients with knee osteoarthritis to guide self-management, provide evidence-based information to patients and physicians and facilitate communication that address patient needs and challenges in disease management. The co-design process with patients, family physicians and researchers, identified a visual symptom graph using PROMs to be amongst the highest priority for functional requirements of the app [17].

In another application of PROMs measures, we have examined the relationship of appropriateness and patient reported outcomes at one-year post-surgery in a prospective cohort study implemented at the two highest volume centres in Alberta which use a centralised intake process for surgical referrals. With the continued increase in rates of arthroplasty surgery, particularly in patients at younger ages and with higher levels of functioning and lower levels of pain, there are concerns about the appropriate selection of candidates for surgery [18, 19]. A key factor for appropriateness suggests that patients should have realistic expectations for total knee arthroplasty (TKA) outcomes. We found that the pre-operative expectations of patients regarding kneeling and psychological wellbeing were significantly associated with the patient-reported level of TKA satisfaction at one year post-surgery [20].

Building on this appropriateness research, another related application of PROMs at the micro level is the role of PROMs to inform setting realistic expectations for patients, and promoting shared decision-making with their care provider [21]. We evaluated whether routinely collected pre- and post-TKA PROMs could be integrated into a patient decision aid to better inform these appropriateness criteria in a randomized controlled trial with a primary outcome measure of decision quality [22]. We found that decision quality improved for patients who completed the decision aid. [Under review].

At the meso level, healthcare providers can use aggregate PROMs data to identify certain outcomes or symptoms in groups of patients or for a clinic site, and compare these data across providers, clinics, or hospital sites. Using the ABJHI Health Data Repository, in the context of the clinical care pathway and a measurement framework with key performance indicators (waiting time, length of surgery, post-surgery length of hospital stay, serious complications, readmissions, a range of PROMs, and patient satisfaction), continuous quality improvement reports are provided routinely to individual health care providers, public health agencies, hospitals and clinics on their performance regarding hip and knee arthroplasty at all of the 14 hospitals that perform hip and knee arthroplasty across the province. These data are used for routine monitoring and surveillance by ABJHI as part of their continuous quality improvement program at the provincial level through Alberta Health Services and the Bone and Joint Health Strategic Clinical Network and the clinic level for all hip and knee clinics in Alberta to inform performance against benchmarks, and at the individual surgeon level to inform patient management [23–25]. Despite the desire to provide real-time feedback of PROMs during the clinical encounter, several technological barriers complicate the integration of web-based data capture with multiple EMR systems across multiple locations in near real-time.

At the macro level, PROMs data can be used to evaluate the performance of the healthcare system to compare outcomes across different jurisdictions or over time and support health policy decision making. Combined with administrative databases as a key source for estimating the burden and epidemiologic trends of chronic diseases such as osteoarthritis [26], and simulation models that reflect how patients transition through the continuum of care from disease onset through end-stage care [27], PROMs data can provide insight into the magnitude of the patient population and burden of disease, healthcare resource requirements and associated healthcare costs. Such tools can be used for estimating how many patients will need care, the nature of that care, and when the care will be needed. This is essential for planning healthcare resource allocation at a system level. These are powerful tools when applied to inform decision making for health care delivery that consider not only the technological aspects of effective care, but also the healthcare context – including the people and processes for the delivery of care [28, 29]. As part of their continuous quality improvement program noted above, ABJHI also reports these data at the macro provincial level through Alberta Health Services and the Bone and Joint Strategic Clinical Network [24, 25]. For instance, PROMs data are being used to develop appropriateness criteria for same-day discharge and accelerated discharge to improve patient throughput and address demand backlog [30]. As examples of how PROMs and PREMs have had an impact, we have seen protocol changes around analgesic use (e.g. elimination of intravenous morphine) due to patient-reported post-op nausea and vomiting reported via PREMS. We have also seen changes in communication methods to address patient-reported deficiencies in coordination of care (e.g. whiteboards in room with discharge planning). Consequently, changes in education and awareness have led to reduced variability in selection of patients for surgery over time (i.e. reduced variance in pre-op WOMAC scores over time).

## Challenges

We have met some general challenges that other PROMs programs might also expect to experience. The transition from the EQ-5D-3L to the 5L was performed gradually between 2013 and 2016. To minimize expense when the paper copies of EQ-5D-5L questionnaires were sent to each clinic, the 3L version questionnaires had to be exhausted before the clinics were willing to deploy the 5L survey. The two versions were in mixed-use for a period, and some patients were administered different versions throughout their care journey. This introduced difficulties in data analysis and interpretation, as the 3L scores had to be up converted to 5L values.

In addition, the integration of electronic data capture (EDC) into clinic workflow remains one of the greatest challenges in PROMs administration. Though some clinics provide patients with a web URL to complete their surveys in advance of the appointment, many patients only complete their surveys at the clinic. This can introduce delays in clinic flow including patients requiring additional time to complete their surveys while in the waiting or exam room, additional staff time required to set up technology for patients to use and to address any patient queries regarding the survey questions, and gaps in data collection when patients abandon surveys before completion due to late arrival at the clinic or faster than expected wait time for the appointment. The transition from paper to EDC highlighted the implementation challenges when deploying at provincial scale. The critical success factors that eased adoption of EDC were a) the availability of staff to assist patients with the technology, b) availability of WIFI connectivity, and c) sufficient or dedicated space for the patients to use while completing their PROMs. Furthermore, patient follow-up processes vary across clinics, with some using automated reminders for patients to complete outstanding surveys, while other clinics are unwilling to rely on technology to support electronic data capture due to limited availability of technologically proficient staff or IT support. Moreover, while some small clinics have staff capacity to follow up with patients to complete their paper surveys, this would overwhelm some other clinics. Although guidelines suggest that 1 year post-operatively is most appropriate for collecting PROMs and PREMs [31], in practice, we included data collection at 3 months post-operatively. With the ever-present pressure to improve efficiencies, some clinics no longer routinely conduct face-to-face follow up appointments at 12 months, which reduces data capture rates. Since most of the functional improvement is achieved by 3-months post-surgery, it has become standard practice in the province to focus analysis and reporting on 3-month results using the substantially larger sample sizes at 3-months.

However, one of the greatest challenges that we faced was building a culture of evidence-based decision making. A critical aspect of the pathway implementation was the establishment of provincial committees to oversee the adoption of new evidence, including the Hip and Knee Arthroplasty Clinical Committee that maintains the clinical standards of care and the Hip and Knee Replacement Working Group that operationalizes the standards into clinical practice. These committees review more than a dozen key performance indicators, including aggregate pre- and post-surgery PROMs results to identify opportunities for improvement, and craft targeted quality improvement initiatives to investigate and address gaps in quality of care.

Based on our experiences, we suggest the following when considering implementation, collection and reporting on the use of PROMs and PREMs in routine clinical practice, our experiences in Alberta have highlighted some important aspects to consider, and we recommend the following:

- Carefully consider the context in which data collection will occur – e.g. if using electronic data collection technology, be sure to confirm that there is reliable and secure WIFI connectivity everywhere;
- Be sure that the clinic staff are familiar and comfortable with the technology before implementation and are able to assist patients in completing the PROMs and PREMs instruments if needed;
- Conduct adequate testing of the data collection instruments with patients to ensure both functionality and usability of the instruments;
- Consider implementation of PROMs and PREMs instruments as a package in one step rather than slow, phased deployments to minimize transition time when making changes (e.g. one instrument followed by another);
- Tailor reports to the audience (clinician, clinic, health system) and include context against benchmarks and trends over time to inform changes in practice.

## Further plans for PROMs applications in other areas of bone and joint health

The implementation of the new model of care for hip and knee arthroplasty has had significant impact on care delivery across the province in terms of multiple dimensions of quality of care including improvements in quality of life post-surgery (effectiveness), satisfaction with overall health care experience (acceptability), appropriateness of resources (appropriateness), reduced length of stay (efficiency), and reduced wait times (accessibility) [32]. The application of PROMs in hip and knee arthroplasty has served as a reference example of at the national and international levels [1, 33]. Furthermore, at a time when wait times continue to grow due to an aging population and unforeseen demands for acute care on the healthcare system, long delays to receive elective surgery such as hip and knee arthroplasty will continue to have negative consequences on patient outcomes that need to be measured and reported to inform health policy [30]. The learnings about the key elements of success for the successful implementation of models of care to reduce waiting times are relevant and transferable to other areas of care delivery [34].

The addition of PROMs data in clinical repositories and analyses enables the system to identify and address issues of inequity, appropriateness, and to explicitly recognize the trade-offs that are inherent in any resource-constrained system. For instance, the ability to empirically quantify the impact of waiting for service on patient quality of life elevates the discussions that are possible to include society’s “willingness to wait” and the appropriateness of global access benchmarks.

## Data Availability

Not applicable.

## Abbreviations

ABJHI: Alberta bone and joint health institute
HUI3: Health Utility Index Version 3
OCR: Optical character recognition
OECD: The organization for economic co-operation and development
PROMs: Patient-reported outcome measures
SF-36: Short Form 36
TKA: Total knee arthroplasty
WOMAC: Western Ontario and McMaster Universities Osteoarthritis Index
